# National Medical Union

**Published:** 1914-02-07

**Authors:** Percy C. Raiment

**Affiliations:** General Secretary, N.M.G.


### National Medical Union.

Sir,—I have perused with much pleasure your article on the National Medical Union, and I most heartily support the proposition that all members of the National Medical Union should purchase Mr. Gibbon's work on Medical Benefit in Germany. The appendices are most valuable, and I have made of them a careful study, but nowhere can I find any justification for the statement that the formation of the Leipziger Verband was in any way analogous to the present National Medical Union. The Leipzig Union is a trade union, and adopts all the methods of ordinary trade unions, particularly in the direction of strikes and strike pay. How, then, can the National Medical Union claim to be in any way analogous to this body when in the same column I find Dr. Skardon Prowse saying: "The National Medical Union since its inception has been opposed to the idea of trade unionism."

Possibly the two latest converts from trade unionism, Messrs. Carswell and Dorrell, will tender an explanation. In the meantime I sincerely hope that the members will read the book recommended to them.—I am, etc.,

Percy C. Raiment, General Secretary, N.M.G.

P.S.—At the meeting of the National Medical Guild in Manchester the actual number present was sixty-three.

